# The Erie County Medical Society and the New Code

**Published:** 1883-02

**Authors:** 


### The Erie County Medical Society and the New Code.

The annual meeting of the Erie County Medical Society was looked forward to with interest, as the occasion upon which the society would have to express itself upon the subject of the New Code. The matter was brought up at the June meeting, by the introduction of a resolution approving of the New Code. The matter was then warmly discussed, and an unexpected leaning toward the support of the resolution was manifest on the part of many members. The upshot at the time was the passage of a resolution to lay on the table. Upon calling up the resolution at the last meeting, however, no attempt was made to sustain it, its advocates undoubtedly despairing of success in supporting it, but expecting to accomplish all they could hope for by defeating the proposal to "instruct" the delegates. The quotation from Addison, "where liberty is gone life grows insipid and has lost its relish," which C. R. Agnew used as a text for his paper, entitled, "Limits of Medical Ethics," we have no doubt had as much weight in influencing certain minds, unable to distinguish between liberty and license, as the specious arguments which make up his paper; and so here, too, a "Bawl for Liberty" arose from every advocate of the New Code present at the meeting. The "injustice" of placing any restriction upon the personal freedom of our delegates was dwelt upon, and the delegates present expressed themselves extremely unwilling to be "instructed." As the majority of them have already ranged themselves among the opponents of the American Code, this was not at all surprising. The action of the society in refusing to instruct leaves all of its delegates at perfect liberty to vote in accordance with their personal convictions, notwithstanding the resolution of mild disapproval of the New Code which was adopted.

